# *In vitro* enzymatic hydrolysis for xylo-oligosaccharide release from wheat-, maize-, maize and wheat distillers dried grains with solubles- and sorghum-based broiler diets

**DOI:** 10.1016/j.psj.2025.106236

**Published:** 2025-12-11

**Authors:** Eunjoo Kim, Andrew Wallace, Mingan Choct, Anna Fickler, Leon Hall, Nishchal K. Sharma

**Affiliations:** a School of Environmental and Rural Science, University of New England, Armidale, NSW, 2350, Australia; b BASF SE, 67056 Ludwigshafen, Germany; c Poultry Hub Australia, University of New England, Armidale, NSW, 2350, Australia

**Keywords:** Broiler, Carbohydrase, In vitro digestion model, Non-starch polysaccharide, Xylo-oligosaccharide

## Abstract

The prebiotic effects of xylo-oligosaccharides (XOS) in poultry are well documented, but their detailed formation during xylan hydrolysis in the gastrointestinal tract remains unclear. This is due mainly to complex interactions among exogenous enzymes used, gut microbiota and the structures of in-feed xylans (including arabinoxylans). This study employed a two-step *in vitro* digestion model simulating the chicken gastrointestinal environment to evaluate XOS production via enzymatic hydrolysis using a xylanase and β-glucanase (XG) preparation across four broiler diets based on wheat, maize, sorghum or maize-wheat distiller’s dried grain with solubles (DDGS). To gain insight into the structural accessibility of xylan-based polysaccharides in diets, xylan profiling was conducted. Xylan profiling showed that the total xylan content was highest in the maize-wheat DDGS-based diet, and lowest in the sorghum-based diet. The wheat-based diet was intermediate, with higher levels than maize, but lower than the maize-wheat DDGS-based diet. In the wheat-based diet, XG increased (*P* < 0.05) xylobiose (XOS2: 8.3 to 13.7 mg/kg), xylotriose (XOS3: 2.0 to 5.2 mg/kg), xylotetraose (XOS4: 1.2 to 2.6 mg/kg), and xylopentaose (XOS5: 0.7 to 1.6 mg/kg). In the maize-based diet, XG increased (*P* = 0.006) XOS2 (3.1 to 3.9 mg/kg), while XOS3 (0.7 mg/kg) remained unchanged; XOS4 and XOS5 were undetected. In the maize-wheat DDGS-based diet, XG markedly increased (*P* < 0.001) XOS2 (21.4 to 283.9 mg/kg), XOS3 (26.4 to 227.9 mg/kg), and XOS4 (8.6 to 22.8 mg/kg), while XOS5 (2.9 mg/kg) was unaffected. In the sorghum-based diet, XG increased (*P* < 0.001) XOS2 (7 to 10.6 mg/kg), while XOS3 (0.9 mg/kg) remained unchanged; XOS4 and XOS5 were undetected. These findings provide *in vitro* evidence that XG can release mid-length XOS from wheat- and maize-wheat DDGS-based diets, supporting their potential to enhance gut health in broilers.

## Introduction

Xylo-oligosaccharides (**XOS**) are short-chain carbohydrates derived from the enzymatic hydrolysis of xylan, a major hemicellulosic component of the plant cell wall (Aachary and Prapulla, 2011; Broekaert et al., 2011). Structurally, XOS vary in chain length, substitution pattern and degree of polymerisation (**DP**), with mid-length oligomers such as xylotriose (**XOS3**), xylotetraose (**XOS4**) and xylopentaose (**XOS5**) gaining interest due to their functional properties in chickens (Morgan et al., 2020). These oligomers have been investigated as emerging feed supplements as prebiotics, selectively stimulating beneficial gut microbiota and short-chain fatty acid production while suppressing pathogens in chickens and humans (Chen et al., 2016; Amorim et al., 2020; Ribeiro et al., 2018).

XOS can be generated from arabinoxylan-rich materials using endo-xylanases, often with ancillary debranching enzymes targeting arabinose-substitutions on the xylan backbone (Broekaert et al., 2011). The efficiency and profile of XOS released are influenced by the interaction between enzyme characteristics (e.g. substrate specificity, mode of action and tolerance to gut conditions) and substrate properties (e.g. degree of substitution, solubility and cross-linking with other cell wall components). In the context of poultry nutrition, these factors collectively determine not only the yield of XOS generated during the enzyme-mediated xylan hydrolysis, but also their size distribution and potential functional effects in the gastrointestinal tract. The DP of XOS plays a critical role in determining their site of fermentation, the persistence of their effects along the gastrointestinal tract, and the resulting short-chain fatty acids (**SCFA**) profile. Longer-length XOS are relatively resistant to degradation in the upper gut and reach the hindgut intact, where they are fermented more slowly and tend to favour butyrate production, a SCFA linked to gut barrier integrity and colonocyte energy supply (Broekaert et al., 2011; Wang et al., 2024). In contrast, short-chain XOS (e.g. xylobiose) are fermented relatively fast, often in the proximal gut, leading to higher lactate and acetate production.

Although the benefits of XOS supplementation in broiler chicken diets are well documented (Morgan et al., 2024; Naeem et al., 2025), the potential for *in situ* XOS release via enzymatic hydrolysis during digestion is less explored, particularly across diverse feed substrates (Craig et al., 2020). Cereals and by-products such as wheat, maize and wheat distiller’s dried grain with solubles (**DDGS**) are commonly used in broiler chicken feed and differ markedly in xylan content and structural complexity. As poultry lack endogenous enzymes to efficiently degrade feed non-starch polysaccharides (**NSP**), the use of supplemental endo-xylanases as feed additives has become a routine practice in poultry production, primarily to improve nutrient utilisation. Beyond this primary role, xylanase can also promote the release of oligosaccharides *in vivo* when suitable substrates are present. For instance, Kim et al. (2022) reported increased levels of oligosaccharide-bound xylose and arabinose in the ileum of broiler chickens fed xylanase-supplemented wheat-based diets, suggesting the enzymatic breakdown of xylans into XOS and/or arabinoxylo-oligosaccharides (**AXOS**) *in vivo*. However, measuring only the monomeric sugar components of oligosaccharides in the ileum provides limited insight into their DP and consequently their potential functional roles in the gut.

To assess the XOS-forming potential of practical feed ingredients under physiological conditions of chickens, reliable approaches are required. The conventional NSP assay developed by Englyst et al. (1994) relies on harsh acid hydrolysis to breakdown crystalline cellulose, which may compromise other constituents and cannot differentiate between enzyme-accessible and resistant xylan fractions. Instead, in the present study, a refined profiling method was applied using pure exo- and endo-xylanases for better prediction of feed xylan susceptibility to enzymatic degradation.

This study evaluated XOS release from four cereal-based broiler feeds based on wheat, maize, maize-wheat DDGS or sorghum using a two-step *in vitro* digestion model simulating broiler gut conditions. A commercial xylanase and β-glucanase preparation (**XG**) was applied to assess the extent and profile of XOS released from NSP-rich substrates and to better understand their potential for *in situ* prebiotic oligosaccharide generation. We hypothesised that XOS release by supplemental enzyme is substrate-dependent, driven by enzyme-accessible xylans identified by xylan profiling, and that this approach would provide a clearer indication of XOS yield and size distribution than conventional NSP assays, which rely on bulk soluble NSP rather than the specific xylan fractions available to the enzyme.

## Materials and methods

### Diets and experimental design

The four test diets reflected common broiler feed formulations and were based on wheat, maize, maize-wheat DDGS or sorghum. All diets were free of supplemental carbohydrate-degrading enzymes and cold-pelleted at 65°C. The ingredient and nutrient composition of the test diets are presented in Table 1. Each test diet was ground to pass through a 0.5 mm sieve and then subjected to a two-step *in vitro* digestion model, with or without a commercial XG preparation (Natugrain® TS, BASF SE, Germany), applied at 560 TXU/kg and 250 TGU/kg of xylanase and β-glucanase, respectively. One thermostable endo-xylanase unit (TXU) is defined as the amount of enzyme that releases 5 micromoles of reducing sugars, measured as xylose equivalents per minute from a buffer solution containing 1 g of arabinoxylan per 100 mL at pH 3.5 and 40°C. One thermostable endo-glucanase unit (TGU) is defined as the amount of enzyme that releases 1 micromole of reducing sugars, measured as glucose equivalents per minute from a buffer solution containing 0.714 g β-glucan per 100 mL at pH 3.5 and 40°C.

**Table 1:** Ingredient and nutrient composition of the test diets (% as-is).

Item, % (as-is)	Wheat-based finisher diet	Maize-based finisher diet	Sorghum-based grower diet	Maize-wheat DDGS-based grower diet
Wheat	59.8	-	-	-
Maize	-	59.1	-	57.1
Sorghum	-	-	63.5	-
Wheat DDGS	-	-	-	18.7
Wheat bran	10	10	-	-
Soybean meal	25.3	25.8	28.2	19.6
Canola oil	2.7	2.7	3.9	0.89
L-Lysine HCl	-	-	0.27	0.43
D, L-Methionine	0.15	0.16	0.35	0.29
L-Threonine	-	-	0.15	0.14
L-Valine	-	-	0.02	0.06
L-Arginine	-	-	0.11	0.22
Choline chloride 60%	0.06	0.11	0.13	0.08
Vitamin premix^1^	0.09	0.09	0.09	0.08
Mineral premix^2^	0.11	0.11	0.11	0.10
Sand	0.42	0.42	0.42	0.02
Titanium dioxide	-	-	0.50	0.50
Limestone	1.05	1.03	1.14	1.06
Salt	0.32	0.32	0.17	0.05
Sodium bicarbonate	-	-	0.36	0.24
Dicalcium phosphate	0.07	0.22	0.53	0.37
Phytase^3^	0.01	0.01	0.01	0.01
*Calculated values, %*				
Dry matter	90.7	89.1	90.0	87.8
AMEn, kcal/kg	3094	3094	3083	2980
Dig Lys	0.95	0.95	1.11	1.04
Dig Met	0.42	0.44	0.61	0.54
Dig Met+Cys	0.74	0.74	0.88	0.79
Dig Thr	0.63	0.65	0.74	0.70
Dig Val	0.88	0.88	0.85	0.79
Dig Iso	0.79	0.78	0.77	0.68
Dig Arg	1.26	1.23	1.20	1.13
Dig Trp	0.25	0.22	0.21	0.19
Calcium	0.74	0.74	0.80	0.80
Available Phosphorus	0.37	0.37	0.40	0.40
Sodium	0.16	0.16	0.18	0.18
Choline, mg/kg	1550	1550	1600	1200

1 Vitamin premix per kg diet (UNE VM, Rabar Pty Ltd): vitamin A, 12 MIU; vitamin D, 5 MIU; vitamin E, 75 mg; itamin K, 3 mg; nicotinic acid, 55 mg; pantothenic acid, 13 mg; folic acid, 2 mg; riboflavin, 8 mg; cyanocobalamin, 0.016 mg; biotin, 0.25 mg; pyridoxine, 5 mg; thiamine, 3 mg; antioxidant, 50 mg.

2 Mineral premix per kg diet (UNE TM, Rabar Pty Ltd): Cu, 16 mg as copper sulfate; Mn, 60 mg as manganese sulfate; Mn, 60 mg as manganous oxide; I, 0.125 mg as potassium iodide; Se, 0.3 mg as sodium selenite; Fe, 40 mg as iron sulfate; Zn, 50 mg as zinc oxide; Zn, 50 mg as zinc sulfate.

3 Natuphos E 10000 G (BASF SE, Germany); AMEn, apparent metabolizable energy corrected for N retention.

### Analysis of non-starch polysaccharides in diets

The Englyst method was used to quantify soluble and insoluble NSP, along with free sugars, in test diets by enzymatically removing starch and protein, and hydrolysing NSP into constituent monosaccharides (Englyst et al., 1994). Briefly, feed samples were defatted using hexane and dissolved in 80 % ethanol/water (v/v) to extract free sugars. The ethanol extract (supernatant) was retained for separate quantification of free sugars. The remaining residue was treated sequentially with thermostable α-amylase (A3306-10ML, Merck, Bayswater, VIC, Australia) and amyloglucosidase (10115-1G-F, Merck, Bayswater, VIC, Australia) to remove starch. Following starch hydrolysis, the sample was precipitated in 80 % ethanol/water (v/v) to separate the soluble NSP fraction (in the supernatant) from the insoluble NSP (in the pellet). To release monosaccharides, soluble NSP fractions were hydrolysed using 2 M trifluoroacetic acid (**TFA**) at 120°C. Insoluble NSP fractions were first solubilised in 12 M sulphuric acid, followed by dilution to 1 M and hydrolysis at 100°C. After acid hydrolysis, samples were neutralised, and the monosaccharides were converted to alditol acetates and analysed by gas chromatography-mass spectrometry (**GC-MS**). The total NSP content was calculated as the sum of individual monosaccharides from both soluble and insoluble fractions, excluding free sugars.

### Xylan and arabinoxylan profiling in diets

**Fig. 1:**
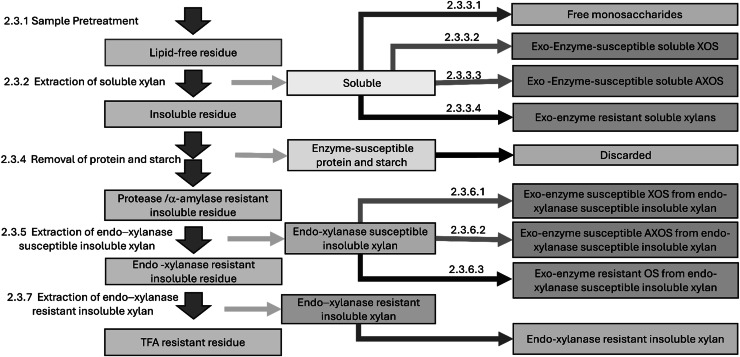
Sequential extraction and hydrolysis workflow used to profile xylan subclasses. The figure shows the workflow with numbered subsections corresponding to the methods.

Xylan subclasses were profiled using a sequential extraction and hydrolysis workflow (Fig. 1), which separates soluble and insoluble fractions and further resolves exo- and endo-enzyme–susceptible and resistant xylans.

### Sample pretreatment

Approximately 200 mg of sample was weighed into a 10 mL centrifuge tube. Any possible endogenous enzymes were inactivated by adding 1.5 mL of 80 % ethanol/water, sealing the tube, and heating at 120°C for 1 h. After cooling, ethanol was removed under nitrogen at 60°C. Once cooled to room temperature, 8 mL of petroleum spirit was added for lipid extraction. Samples were vortexed, mixed on a rotary mixer for 15 min and centrifuged at 2500 x g for 10 min. The supernatant was discarded, and extraction was repeated with another 8 mL of petroleum spirit. The remaining solvent was evaporated under nitrogen at 40°C, and samples were vacuum-dried overnight.

### Extraction of soluble xylan

Soluble xylans were extracted by adding 3 mL of 50 % ethanol/water to the sample, followed by mixing and centrifugation. The supernatant was decanted into a clean 10 mL volumetric flask. This step was repeated twice, the extracts (approximately 8.5 mL) were combined and adjusted to 10 mL with 50 % ethanol/water. A 1 mL aliquot was transferred to a 2 mL microtube, evaporated under nitrogen at 60°C, and reconstituted in 1 mL of water containing 0.05 % NaN₃ for downstream soluble xylan hydrolysis. The remaining insoluble residue was vacuum-dried and used for subsequent insoluble xylan extractions.

### Selective hydrolysis of soluble xylans

The soluble extract was divided and subjected to four sequential treatments of increasing hydrolytic intensity. These treatments were designed to target: free monosaccharides (Fig. 1, 2.3.3.1); exo-enzyme susceptible soluble XOS (Fig. 1, 2.3.3.2); exo-enzyme susceptible soluble AXOS (Fig. 1, 2.3.3.3); and exo-enzyme resistant soluble xylans (Fig. 1, 2.3.3.4). Hydrolysis products from each treatment were subsequently subjected to GC-MS analysis and used for calculation of the respective xylan-derived fractions.

### Free monosaccharides

An aliquot (50 μL) of soluble extract was mixed with 50 μL of 200 ppm d-xylose-¹³C₅ as internal standard (Novachem Pty Ltd, Collingwood, VIC, Australia) in a 10 mL centrifuge tube. The mixture was evaporated to dryness under a steady stream of nitrogen at 75°C, and the residues were analysed by GC-MS. The concentration of soluble free monosaccharides was measured directly.

### Exo-enzyme-susceptible soluble XOS

An enzyme solution selective for XOS was prepared by diluting 50 μL of β-xylosidase (E-BXSR-1KU, Megazyme, Wicklow, Ireland) in 1450 μL of 200 mM sodium acetate buffer (pH 5.0). Reaction mixtures contained 50 μL of soluble extract, 50 μL of the internal standard, and 50 μL of enzyme solution in a 10 mL centrifuge tube. Samples were incubated at 40–50°C for 1 h, dried under nitrogen at 75°C, and analysed by GC-MS. The concentration of exo-enzyme susceptible soluble XOS was calculated by subtracting the soluble free monosaccharides (Fig. 1, 2.3.3.1) from the products released by β-xylosidase hydrolysis.

### Exo-enzyme-susceptible soluble AXOS

An enzyme solution selective for both XOS and AXOS was prepared by combining 50 μL of β-xylosidase and 50 μL of α-arabinofuranosidase (E-AFASE, Megazyme, Wicklow, Ireland) in 1400 μL of 200 mM sodium acetate buffer (pH 5.0). Reaction mixtures contained 50 μL of soluble extract, 50 μL of the internal standard, and 50 μL of enzyme solution. Samples were incubated at 40–50°C for 1 h, dried under nitrogen at 75°C, and analysed by GC-MS. The concentration of exo-enzyme susceptible soluble AXOS was calculated by subtracting both the soluble free monosaccharides (Fig. 1, 2.3.3.1) and the exo-enzyme susceptible soluble XOS (Fig. 1, 2.3.3.2) from the products released by the combined β-xylosidase and α-arabinofuranosidase hydrolysis.

### Exo-enzyme resistant soluble xylans

Aliquots of soluble extract (50 μL) were combined with 50 μL of the internal standard and 20 μL of TFA in a 10 mL centrifuge tube. Tubes were sealed and heated at 115°C for 1 h, cooled to below 75°C, and centrifuged at 2500 × *g* for 2 min. Water and TFA were evaporated under nitrogen at 75°C, and the residues were analysed by GC-MS (Fig. 1, 2.4). The concentration of exo-enzyme resistant soluble xylans was calculated by subtracting the soluble free monosaccharides (Fig. 1, 2.3.3.1), exo-enzyme susceptible soluble XOS (Fig. 1, 2.3.3.2), and exo-enzyme susceptible soluble AXOS (Fig. 1, 2.3.3.3) from the total products released by TFA hydrolysis.

### Removal of protein and starch

The dried insoluble residue was suspended in 4 mL of water (containing 0.05 % NaN_3_), and 500 μL of 0.5 M sodium phosphate buffer (pH 7.5) was added, followed by treatment with 20 μL of protease solution (E-BSPRT-40ML, Megazyme, Wicklow, Ireland). Samples were incubated at 50–60°C for 12 h, centrifuged at 2500 × *g* for 10 min, and the supernatant discarded. The solid residue was washed twice with 3 mL of water, each wash consisting of resuspension, centrifugation, and removal of the supernatant.

For starch removal, the insoluble residue was resuspended in 4 mL of water, heated at 100°C for 1 h to gelatinise the starch, cooled, and treated with 20 μL of α-amylase solution (10065-10 G, Merck, Bayswater, VIC, Australia) at 10 mg/mL in 200 mM sodium acetate buffer (pH 6). Samples were incubated at 40°C for 12 h, centrifuged at 2500 × *g* for 10 min, and the supernatant discarded. The solid residue was washed twice with 3 mL of water, using the same suspension, centrifugation, and decanting steps. The final residue was vacuum-dried overnight and used for determination of endo-xylanase susceptible insoluble xylans (Fig. 1, 2.3.5).

### Extraction of endo-xylanase susceptible insoluble xylans

To extract endo-xylanase susceptible insoluble xylans, the dried residue was resuspended in 4 mL of water (0.05 % NaN_3_) and treated with 100 μL of endo-xylanase solution (X2753-10 G, Merck, Bayswater, VIC, Australia) at 10 mg/mL in 200 mM sodium acetate buffer (pH 5). The mixture was incubated with shaking at 40°C for 12 h, centrifuged at 2500 × *g* for 10 min, and the supernatant collected. This supernatant contained soluble XOS, AXOS, and other xylan-derived oligosaccharides generated by selective endo-xylanase hydrolysis and was used for further enzymatic hydrolysis (Fig. 1, 2.3.6). The solid residue was washed twice with 3 mL of water, vacuum-dried, and used for subsequent TFA hydrolysis (Fig. 1, 2.3.7).

### Selective hydrolysis of endo-xylanase extracts

The supernatant from endo-xylanase digestion (Fig. 1, 2.3.5) was divided into three aliquots and subjected to differential hydrolysis, analogous to the treatments applied to soluble xylans (Fig. 1, 2.3.3). The aliquots were used to determine endo/exo-enzyme susceptible xylans (Fig. 1, 2.3.6.1), endo/exo-enzyme susceptible arabinoxylans (Fig. 1, 2.3.6.2), and endo/exo-enzyme resistant xylans (Fig. 1, 2.3.6.3). Hydrolysates were dried and analysed by GC-MS, and the results were used for calculation of the respective xylan-derived fractions.

### Exo-enzyme susceptible XOS from endo-xylanase susceptible insoluble xylan

Hydrolysis was carried out as described for soluble exo-enzyme susceptible XOS (Fig. 1, 2.3.3.2), using 50 μL of the endo-xylanase extract as substrate. Products were analysed by GC-MS, and the concentration of endo/exo-enzyme susceptible xylans was measured directly.

### Exo-enzyme susceptible AXOS from endo-xylanase susceptible insoluble xylan

Hydrolysis was carried out as described for soluble exo-enzyme susceptible AXOS (Fig. 1, 2.3.3.3), using 50 μL of the endo-xylanase extract as substrate. Products were analysed by GC-MS, and the concentration of endo/exo-enzyme susceptible arabinoxylans was calculated by subtracting the endo/exo-enzyme susceptible xylans (Fig. 1, 2.3.6.1).

### Exo-enzyme resistant oligosaccharides from endo-xylanase susceptible insoluble xylan

Hydrolysis was carried out as described for soluble exo-enzyme resistant xylans (Fig. 1, 2.3.3.4), using 50 μL of the endo-xylanase extract as substrate. Products were analysed by GC-MS, and the concentration of endo/exo-enzyme resistant xylans was calculated by subtracting both the endo/exo-enzyme susceptible xylans (Fig. 1, 2.3.6.1) and the endo/exo-enzyme susceptible arabinoxylans (Fig. 1, 2.3.6.2).

### Endo-xylanase resistant insoluble xylan

The insoluble residue obtained after endo-xylanase extraction was resuspended in 3000 μL comprising 1500 μL of water and 1500 μL of internal standard (200 ppm d-xylose-¹³C₅). Then, 600 μL of TFA was added, and the samples were sealed and heated at 120°C for 1 h, cooled, and centrifuged at 2500 × *g* for 10 min. An aliquot of 120 μL of the supernatant was transferred into a 10 mL centrifuge tube, dried under a steady stream of nitrogen at 75°C, and analysed by GC-MS. The concentration of endo-xylanase resistant insoluble xylan was measured directly.

### Xylan and NSP analysis by gas chromatography–mass spectrometry

Xylose and arabinose released from xylan and NSP samples were identified and quantified by GC-MS as their alditol acetate derivatives, adapted from previously published methods (Blakeney et al., 1983). Samples were reduced with 25 μL of 3 M ammonia and 2 % NaBH₄ in 200 μL of dimethyl sulfoxide at 40°C for 90 min. The reaction was quenched with 20 μL of acetic acid and mixed thoroughly. After cooling, 40 μL of 1-methylimidazole and 400 μL of acetic anhydride were added for acetylation and reacted at 25°C for 30 min, followed by quenching with 800 μL of water. Derivatives were extracted with 400 μL of dichloromethane, and 200 μL of the lower organic phase was transferred to GC-MS vials containing 800 μL of ethyl acetate.

Analysis was performed using an Agilent 7890A/5977C GC-MS equipped with an HP-5MS column (30 *m* × 250 μm × 0.25 μm) with a 1 μL injection (1:10 split). The oven was held at 170°C for 13 min, ramped to 220°C at 100°C/min, and held for 4.5 min (total run time 18 min). Quantification was carried out in SIM mode, monitoring *m/z* 289.1 for arabinose and xylose and *m/z* 291.1 for the internal standard. Calibration curves were prepared using standards processed by the same derivatisation procedure.

### In vitro digestion

A two-step *in vitro* digestion was performed to simulate the gastric and intestinal phases of the broiler chicken digestive system, adapted from the method described by Morgan et al. (2020). The gastric phase represented the proventriculus and gizzard, while the intestinal phase represented the duodenum and jejunum. Each test diet was ground through a 1 mm screen, and 2 g was weighed into 10 mL tubes, with five replicates per treatment.

For the gastric phase, samples were suspended in 4 mL of water, the pH was adjusted to 3.5 with 1 M HCl, and 50 μL of pepsin solution at 10 mg/mL (1:2500; ChemSupply, Gillman, SA, Australia) was added. For enzyme-supplemented treatments, 20 μL of the commercial enzyme preparation Natugrain® TS, providing 560 TXU/kg xylanase and 250 TGU/kg β-glucanase, was also added. Samples were incubated with shaking at 42°C for 20 min.

For the intestinal phase, immediately after the gastric phase, the pH was adjusted to 6 with 1 M NaHCO₃, and 50 μL of pancreatin (1 x USP) solution at 40 mg/mL (MP Biomedicals Australia Pty Ltd, Seven Hills, NSW, Australia) was added. Samples were incubated with shaking at 42°C for 55 min, followed by a further 5 min without shaking.

To quench enzyme activity, 50 μL of the upper liquid was transferred directly into 400 μL of ethanol. The solution was sealed and heated to 120°C for 30 min to denature enzymes. Samples were then dried under nitrogen at 75°C, reconstituted in 500 μL of internal standard solution (1 ppm d-xylose-¹³C₅), and prepared for XOS determination.

### Determination of XOS by liquid chromatography-mass spectrometry

Aliquots of 200 μL from the *in vitro* digesta were passed through preconditioned Bond Elut C18 end-capped SPE cartridges (50 mg, 1 mL; Agilent Technologies, Santa Clara, CA, USA). To remove residual α-glucans, 50 μL of α-amyloglucosidase solution at 1 mg/mL in 200 mM sodium acetate buffer (pH 5.0) was added and incubated at 50°C for 1 h. XOS were derivatised with 200 μL of 0.5 M 1-phenyl-3-methyl-5-pyrazolone (PMP) in 2 M ammonia at 75°C for 75 min. The reaction was quenched with 3 M formic acid, and excess PMP was removed by three chloroform extractions. PMP-derivatised XOS in the aqueous phase were extracted onto C18 SPE, washed with 1 mL of water, eluted with 40 % acetonitrile in 0.1 % formic acid, and diluted with 0.1 % formic acid to 20 % acetonitrile in 0.1 % formic acid.

Samples were analysed using a Shimadzu liquid chromatography mass spectrometry (**LC-MS**)-8050 equipped with an Agilent Zorbax SB-C18 column (1.8 μm, 3.0 × 150 mm) maintained at 60°C. Isocratic elution was performed with 17 % acetonitrile in water containing 0.1 % formic acid at 0.4 mL/min. XOS2–XOS5 were identified and quantified by comparing retention times and intensities with PMP-derivatised standards prepared from commercial XOS (Megazyme, Wicklow, Ireland) using the same derivatisation procedure. Detection was carried out in positive SIM mode, monitoring the following m/z values: PMP-XOS2 (*m/z* 613.2), PMP-XOS3 (*m/z* 745.3), PMP-XOS4 (*m/z* 877.3), PMP-XOS5 (*m/z* 1009.3) and the internal standard (*m/z* 486.2).

### Statistical analysis

All data were analysed using IBM SPSS Statistics version 29. Independent samples *t*-tests with two-tailed P-values were used to compare the means between treatments. Statistical significance was set at *P* *<* 0.05, while trends towards significance were considered at 0.05 ≤ *P* ≤ 0.10.

## Results and discussion

### NSP and xylan quantification

Based on the Englyst method (Table 2), the wheat-based diet contained the highest total NSP content, followed by the maize-wheat DDGS and maize-based diets, while the sorghum-based diet contained the lowest. Soluble NSP was highest in the maize-wheat DDGS-based diet (14.0 g/kg), while the sorghum-based diet contained only a minimal level (3.0 g/kg). Insoluble NSP was highest in the wheat-based diet (80.4 g/kg), followed closely by the maize-based diet (79.6 g/kg) and lowest in the sorghum-based diet (52.2 g/kg). Xylose and arabinose dominated the insoluble NSP fraction in the wheat- and maize-based diets, suggesting a high presence of insoluble arabinoxylans. The maize-based diet had the highest level of free sugars (21.8 g/kg), primarily from free glucose (17.5 g/kg).

**Table 2:** Non-starch polysaccharide profile of test diets by Englyst method (g/kg DM)^1^.

Item (g/kg DM)	Maize-based	Maize-wheat DDGS-based	Wheat-based	Sorghum-based
Total NSP constituent sugars	86.1	89.0	90.2	54.2
Galactose	12.6	11.1	13.2	10.7
Xylose	29.9	32.7	30.6	9.7
Glucose	29.0	27.3	30.1	24.7
Arabinose	22.7	25.3	24.1	12.6
Mannose	1.5	2.6	2.0	1.6
Ribose	0.1	0.2	0.3	0.2
Rhamnose	0.3	0.3	0.4	0.4
Fucose	0.7	0.6	0.8	0.8
Soluble NSP constituent sugars	6.3	14.0	10.9	3.0
Galactose	1.4	2.3	1.9	0.7
Xylose	0.8	3.7	2.5	0.0
Glucose	3.1	4.6	4.1	2.0
Arabinose	1.4	4.0	3.3	0.4
Mannose	0.2	1.0	0.3	0.1
Ribose	0.1	0.1	0.1	0.1
Rhamnose	ND	ND	ND	ND
Fucose	ND	ND	ND	ND
Insoluble NSP constituent sugars	79.6	75.0	80.4	52.2
Galactose	11.2	8.9	11.1	10.3
Xylose	29.1	29.0	28.7	9.7
Glucose	25.7	22.9	26.5	23.4
Arabinose	21.4	21.1	21.1	12.3
Mannose	1.2	1.6	1.8	1.5
Ribose	0.1	0.1	0.2	0.2
Rhamnose	0.3	0.3	0.4	0.4
Fucose	0.7	0.6	0.7	0.7
Oligosaccharide constituent sugars	21.8	13.4	18.0	13.4
Galactose	4.4	2.7	3.4	3.5
Xylose	0.1	1.7	0.1	0.1
Glucose	17.5	8.4	15.1	11.4
Arabinose	0.3	1.0	0.3	0.3
Mannose	1.8	0.8	1.0	0.9
Ribose	ND	ND	0.1	0.0
Rhamnose	0.2	0.1	0.2	0.2
Fucose	ND	ND	ND	ND

1 Each sample was analysed in triplicate; NSP, non-starch polysaccharides; ND=Not detected; DM, dry matter; DDGS, distillers’ dried grain with solubles.

To gain further insight into the structural accessibility of xylan-based polysaccharides, xylan profiling was conducted. Xylans were extracted and sequentially hydrolysed using purified endo-xylanase, exo-xylanase, α-l-arabinofuranosidase and TFA under optimal conditions to ensure the complete hydrolysis. This method provided additional insights of structural information beyond monomeric constituent sugars quantification by conventional NSP assay. Based on the xylan profiling results (Table 3), total xylan content (i.e., free + xylan-bound xylose and arabinose) was highest in the maize-wheat DDGS-based diet and lowest in the sorghum-based diet. Soluble fractions, likely representing water-extractable XOS and AXOS, were most abundant in the maize-wheat DDGS-based diet, although exo-enzyme resistant fractions constituted the majority of XOS and AXOS across all diets, suggesting that a substantial proportion of cereal xylans are structurally recalcitrant and not readily degraded by exo-acting xylanase (Saulnier et al., 2007; Lu et al., 2025).

**Table 3:** Xylan profiling of test diets^1^.

Item (g/kg)	Maize- based	Maize-wheat DDGS-based	Wheat-based	Sorghum-based
**Total xylan**				
Xylose	29.3	42.4	33.6	12.7
Arabinose	24.8	33.4	28.4	18.8
**Soluble extract**				
Total XOS and AXOS				
Xylose	0.54	5.05	1.34	1.57
Arabinose	1.18	5.40	2.01	1.97
Exo-susceptible XOS				
Xylose	0.04	0.60	0.05	0.03
Exo-susceptible AXOS				
Xylose	0.03	1.13	0.08	0.04
Arabinose	0.15	1.59	0.53	0.15
Exo-resistant XOS+AXOS				
Xylose	0.47	3.32	1.21	1.50
Arabinose	1.03	3.81	1.48	1.82
**Endo-xylanase extract**				
Total xylan				
Xylose	3.14	5.43	12.24	0.43
Arabinose	1.04	1.63	4.21	0.25
Exo-enzyme–susceptible XOS (endo-xylanase extract)				
Xylose	1.19	2.55	5.18	0.06
Exo-enzyme–susceptible AXOS (endo-xylanase extract)				
Xylose	1.00	1.49	3.57	0.04
Arabinose	0.41	0.71	1.69	0.05
Exo-enzyme–resistant xylans (endo-xylanase extract)				
Xylose	0.95	1.38	3.49	0.33
Arabinose	0.63	0.92	2.52	0.20
**Endo-xylanase–resistant insoluble xylans**				
Xylose	25.60	31.91	19.97	10.72
Arabinose	22.57	26.34	22.21	16.53

1 Each sample was analysed in duplicate; XOS, xylo-oligosaccharides; AXOS, arabinoxylo-oligosaccharides.

The xylanase-extractable fraction is considered the most relevant for *in situ* XOS production. Among the test diets, the wheat-soy diet showed the highest level of xylans (xylose 12.24 g/kg, arabinose 4.21 g/kg), indicating greater susceptibility to endo-xylanase. The maize-wheat DDGS diet showed a moderate release, while the maize and sorghum diets showed very limited hydrolysis, suggesting more recalcitrant xylan structures that are less accessible to enzymatic degradation (Yan et al., 2019). These differences in accessibility align with previous reports showing less consistent responses to xylanase in broilers fed maize or sorghum-based diets compared with wheat-based diets (Selle et al., 2010; Kim et al., 2022). Across all diets, a substantial proportion of xylans remained intact, reflecting limited enzymatic access to tightly bound or highly substituted domains. Because harsh sulphuric acid used in the Englyst method can degrade other NSP and obscure structural features, the xylan profiling approach incorporated a mild TFA step to identify acid-labile xylans not accessible to enzymes. This fraction represents complex or highly substituted structures that are likely to remain intact in the chicken gut. The maize-wheat DDGS diet contained the highest TFA-extractable xylose (31.91 g/kg) and arabinose (26.34 g/kg), indicating a large reservoir of structurally complex xylans that are less responsive to supplemental enzymes under physiological conditions. These findings help explain variable cereal responsiveness to xylanase and highlight the value of structural profiling for predicting prebiotic oligosaccharide release *in vivo*.

Overall, the maize-wheat DDGS diet contained the highest total and soluble xylan and arabinoxylan content, largely due to the inclusion of wheat DDGS. However, much of this fibre was resistant to enzymatic hydrolysis, indicating highly branched or tightly bound structures. In contrast, the wheat-based diet showed the greatest susceptibility to enzymatic degradation under optimal conditions, reflected in its higher endo-xylanase-releasable fractions. This indicates that wheat xylans are more accessible and may explain the stronger broiler responses to xylanase when wheat or its by-products are included in diets. The sorghum-based diet had the lowest xylan content and poorest enzyme susceptibility, while the maize-based diet had moderate xylan levels with limited accessibility.

When comparing conventional NSP analysis (Englyst method) to enzymatic xylan profiling, notable discrepancies emerged. The Englyst method indicated higher soluble NSP in the maize-wheat DDGS diet compared with the wheat-based diet (14.0 g/kg vs. 10.9 g/kg), whereas xylan profiling showed substantially greater xylanase-extractable xylans in the wheat diet (12.24 g/kg xylose and 4.21 g/kg arabinose) than in the maize-wheat DDGS diet (5.43 g/kg and 1.63 g/kg). This demonstrates that soluble NSP measured by Englyst does not necessarily represent the xylan fractions that are actually accessible to xylanase activity. Enzymatic profiling therefore provides a more accurate indication of fibre susceptibility to supplemental hydrolysis and potential for *in situ* prebiotic oligosaccharide release.

### XOS release followed by 2-step *in vitro* digestion

Table 4 presents the concentrations of specific XOS (XOS2-XOS5) released from the maize-based diet following *in vitro* 2-step digestion, with or without XG. XOS2 was the predominant entity and increased by 25.8 % with XG inclusion (from 3.1 to 3.9 mg/kg; *P* = 0.006). XOS4 and XOS5 were not detected, indicating limited partial hydrolysis, which aligns with earlier xylan profiling showing only marginal amounts of xylanase-susceptible xylans in this diet. Despite low substrate accessibility, XG addition still resulted in a modest XOS2 release.

**Table 4:** Xylo-oligosaccharides (mg/kg) released, followed by *in vitro* 2-step digestion of the maize-based diet with or without enzymes^1^.

Diet^2^	XG	XOS5	XOS4	XOS3	XOS2
Maize-based	+	ND^3^	ND	0.7	3.9^a^
	-	ND	ND	0.7	3.1^b^
SEM^4^				0.08	0.17
*P*-value				0.326	0.006

1 Means (n=5) within columns not sharing a common superscript are significantly different (*P* < 0.05);

2 XG, a commercial xylanase + β-glucanase preparation (Natugrain® TS, 560 TXU/kg and 250 TGU/kg of xylanase and β-glucanase, respectively, BASF SE, Germany); XOS, xylo-oligosaccharides.

3 ND, not detected.

4 Pooled standard error of the mean.

Although overall substrate accessibility was low, the consistent increase in XOS2 highlights that XG was still able to act on the small proportion of accessible arabinoxylans within the maize matrix. This indicates that even in maize-based diets, where NSP are tightly bound and poorly extractable (Kim et al., 2022; Kim et al., 2025b), XG can still release small amounts of short-chain XOS when conditions permit. XOS2, a dimer, is rapidly fermented and is typically associated with lactate generation in the hindgut (Zhao et al., 2024), which contrasts with the physiological roles of mid- and longer-chain XOS that support more gradual hindgut fermentation (Gullon et al., 2008; Zhao et al., 2024). In the maize-based diet, XG addition appears to mediate only limited xylan degradation, likely acting on relatively unbranched regions of the xylan backbone, thereby increasing XOS2 without generating XOS3 to XOS5. This pattern is consistent with the restricted solubility and tight cell wall associations of maize NSP. Even so, the detectable rise in XOS2 demonstrates a small but measurable enzymatic response under these conditions.

**Fig. 2:**
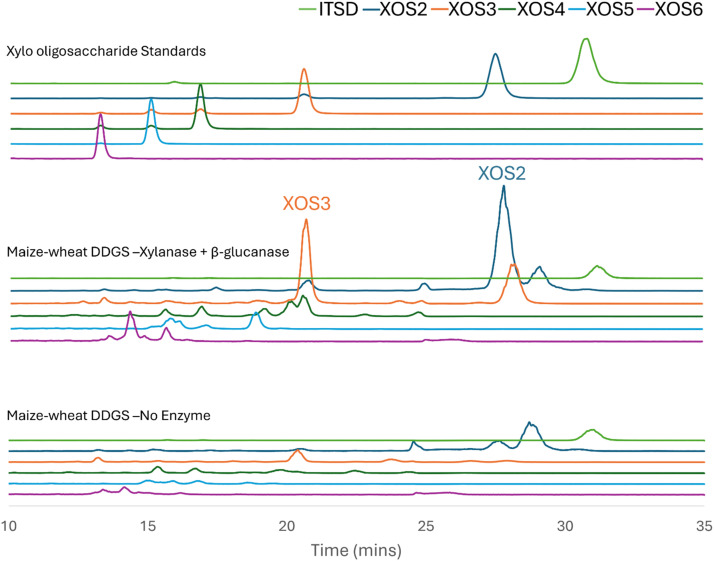
LC-MS chromatograph illustrating xylo-oligosaccharides (XOS2 and XOS3) increase with enzyme treatment in maize-wheat distillers dried grain with solubles (DDGS)-based diet. The enzyme used was a commercial xylanase + β-glucanase preparation (Natugrain® TS, 560 TXU/kg and 250 TGU/kg of xylanase and β-glucanase, respectively, BASF SE, Germany); ITSD is the internal standard.

Table 5 shows the XOS concentrations released from the maize-wheat DDGS-based diet following *in vitro* simulated digestion, with or without the addition of XG. Compared to the control treatment, XG led to a substantial increase in all detected XOS with XOS2 by 13.3-fold (*P* < 0.001), XOS3 by 8.6-fold (*P* < 0.001) and XOS4 by 2.7-fold (*P* < 0.001), with XOS2 being most abundant. Fig. 2 presents the LC-MS chromatograph from one replication illustrating XOS2 and XOS3 increase with and without enzyme treatment. Interestingly, this release of XOS contrasts with the xylan profiling results, which showed relatively low levels of xylanase-extractable xylans in the same diet. These results were likely associated with the highest free XOS and AXOS measured by xylan profiling in the maize-wheat DDGS-based diet among the four test diets. Those free XOS and AXOS were readily available to break down to short chain XOS2 and XOS3. Wheat DDGS production often involves a heat, enzymatic treatment and fermentation of origin wheat during ethanol production and subsequent processing (Pedersen et al., 2014). These physicochemical alterations may have loosened cell wall integrity, subsequently increasing the proportion of XOS and lowering xylanase-susceptible fractions. During simulated digestion, XG addition may have selectively depolymerised these free, less-decorated oligosaccharides into shorter forms (i.e. XOS2 and XOS3), while exhibiting limited activity against the remaining highly branched or acid-labile xylans, as evidenced by this study, where XOS4 and XOS5 releases were only marginal. It is highly likely that the enzymatic action of XG may favour soluble, fewer substitution XOS over tightly bound or structurally complex arabinoxylans.

**Table 5:** Xylo-oligosaccharides (mg/kg) released, followed by *in vitro* 2-step digestion of a maize-wheat DDGS-based diet with or without enzymes 1.

Diet^2^	XG	XOS5	XOS4	XOS3	XOS2
Maize-wheat DDGS-based	+	2.5	22.8^a^	227.9^a^	283.9^a^
	-	2.9	8.6^b^	26.4^b^	21.4^b^
SEM^3^		0.21	2.42	34.17	44.59
*P*-value		0.211	<0.001	<0.001	<0.001

1 Means (n=5) within columns not sharing a common superscript are significantly different (*P* < 0.05).

2 XG, a commercial xylanase + β-glucanase preparation (Natugrain® TS, 560 TXU/kg and 250 TGU/kg of xylanase and β-glucanase, respectively, BASF SE, Germany); XOS, xylo-oligosaccharides.

3 Pooled standard error of the mean.

Table 6 shows the levels of XOS released from the wheat-based diet following *in vitro* digestion with and without XG addition. XG significantly increased (*P* < 0.05) the release of all measured XOS, including XOS2 (*P* = 0.033), XOS3 (*P* = 0.017), XOS4 (*P* = 0.042) and XOS5 (*P* = 0.001). The most abundant XOS was XOS2, increasing from 8.3 mg/kg (–XG) to 13.7 mg/kg (+XG), followed by XOS3 (2.0 to 5.2 mg/kg), XOS4 (1.2 to 2.6 mg/kg) and XOS5 (0.7 to 1.6 mg/kg).

**Table 6:** Xylo-oligosaccharides (mg/kg) released, followed by *in vitro* 2-step digestion of a wheat-based diet with or without enzymes^1^.

Diet^2^	XG	XOS5	XOS4	XOS3	XOS2
Wheat-based	+	1.6^a^	2.6^a^	5.2^a^	13.7^a^
	-	0.7^b^	1.2^b^	2.0^b^	8.3^b^
SEM^3^		0.16	0.37	0.74	1.35
*P*-value		0.001	0.042	0.017	0.033

1 Means (n=5) within columns not sharing a common superscript are significantly different (*P* < 0.05);

2 XG, a commercial xylanase + β-glucanase preparation (Natugrain® TS, 560 TXU/kg and 250 TGU/kg of xylanase and β-glucanase, respectively, BASF SE, Germany); XOS, xylo-oligosaccharides.

3 Pooled standard error of the mean.

When wheat is used as the major energy source in broiler diets, its inclusion often exceeds 50 %, requiring exogenous xylanase to counter the anti-nutritive impacts such as increased digesta viscosity from soluble arabinoxylans. Our findings indicate that xylanase plays a broader role than viscosity reduction. In the wheat-based diet, XG addition led to a proportionally greater increase in XOS4 and XOS5 *in vitro*. This is notable when compared with the maize-wheat DDGS diet, which had the highest pre-existing free XOS yet produced mainly XOS2 with XG. Despite having lower free XOS, the wheat-based diet generated a wider and more functionally relevant range of XOS, including XOS3, XOS4 and XOS5. Especially, XOS3 and XOS4 are of interest in poultry production, as they tend to resist proximal digestion and reach the hindgut, where they support more sustained microbial fermentation. XOS5 is considered particularly beneficial due to its slower fermentation by hindgut bacteria and higher prebiotic potential (Van De Wiele et al., 2007), contributing to butyrate production (De Maesschalck et al., 2015; Zhao et al., 2024). Butyrate supports gut barrier integrity and provides energy to enterocytes, which are important for poultry health and performance. These *in vitro* observations align with xylan profiling, which showed the highest levels of xylanase-extractable xylans in the wheat-based diet, indicating superior substrate accessibility. The same wheat-based diet had also been tested in an earlier *in vivo* broiler study by the same research group (Kim et al., 2025a), where XG supplementation was associated with higher caecal butyrate concentrations than in non-supplemented birds. In vitro XOS2 to XOS5 concentrations from the present study were positively correlated with *in vivo* caecal SCFA production, with XOS5 showing the strongest association (*r* = 0.523, *P* = 0.018, data not presented). Together, the results highlight the capacity of XG to generate functionally relevant XOS from wheat-based diets.

Collectively, the wheat-based diet showed the strongest *in vitro* response to XG, particularly in the release of XOS4 and XOS5. In contrast, the maize-based diet generated very limited XOS, while the maize wheat DDGS diet mainly produced shorter chain forms (XOS2). These patterns align with the xylan profiling results, indicating that both the extent and size of XOS released were dependent on the accessibility and structure of underlying xylan. The findings further show that xylan profiling provides clearer insight into the enzyme accessible xylan fractions and the expected XOS chain lengths, which are not captured by conventional NSP assays.

Table 7 presents the XOS released from the sorghum-soy diet following *in vitro* 2-step digestion, with or without XG inclusion. Only XOS2 and XOS3 were detected. The XOS2 level significantly increased with XG inclusion (*P* < 0.001), indicating that XG facilitated the breakdown of readily degradable XOS into short-chain form. These results confirm that the sorghum-based diet is minimally responsive to xylanase, consistent with earlier xylan profiling showing low total xylan content and poor enzyme accessibility. The absence of mid- and long-chain XOS indicates that sorghum xylans are either tightly bound within cell wall matrices or possess a structural feature that resists enzymatic degradation, thereby limiting their functional potential to result in *in situ* prebiotic XOS release.

**Table 7:** Xylo-oligosaccharides released, followed by *in vitro* 2-step digestion of a sorghum-based diet with or without enzymes^1^.

Diet^2^	XG	XOS5	XOS4	XOS3	XOS2
Sorghum-based	+	ND^3^	ND	0.9	10.6
	-	ND	ND	0.9	7.0
SEM^4^				0.09	0.63
P-value				0.387	< 0.001

1 Means (n=5) within columns not sharing a common superscript are significantly different (*P* < 0.05);

2 XG, a commercial xylanase + β-glucanase preparation (Natugrain® TS, 560 TXU/kg and 250 TGU/kg of xylanase and β-glucanase, respectively, BASF SE, Germany), XOS, xylo-oligosaccharides.

3 ND, not detected.

4 Pooled standard error of the mean.

## Conclusions

This study showed that XG mediated the release of XOS from cereal-based broiler diets *in vitro*, with the extent and pattern of XOS production differing among feed substrates. Wheat- and maize-wheat DDGS-based diets responded most to XG, producing a broader range of XOS (XOS2 to XOS5), whereas maize- and sorghum-based diets generated only small amounts of short-chain XOS, which was consistent with their lower xylan accessibility. Xylan profiling offered useful context for interpreting these responses, demonstrating that conventional NSP values do not necessarily reflect enzyme-releasable fibre. Although Englyst analysis indicated higher soluble NSP in the maize-wheat DDGS diet, xylan profiling showed that the wheat-based diet provided more accessible xylan structures for enzymatic action. These observations highlight the value of characterising fibre structure when assessing enzyme–substrate compatibility. Overall, the findings suggest that xylanase and β-glucanase combinations could support *in situ* generation of XOS in diets containing wheat or wheat by-products, which could contribute to improved fibre utilisation and potentially support gut function. Confirmation of these effects under practical feeding conditions will require further *in vivo* studies, particularly to clarify the roles of specific XOS profiles in shaping microbial activity and the functional benefits on host physiology.

## CRediT authorship contribution statement

**Eunjoo Kim:** Writing – review & editing, Writing – original draft, Methodology, Investigation, Formal analysis, Data curation, Conceptualization. **Andrew Wallace:** Writing – review & editing, Methodology, Investigation, Formal analysis, Data curation. **Mingan Choct:** Writing – review & editing, Funding acquisition, Conceptualization. **Anna Fickler:** Writing – review & editing, Methodology. **Leon Hall:** Writing – review & editing, Methodology. **Nishchal K. Sharma:** Writing – review & editing, Writing – original draft, Supervision, Methodology, Investigation, Conceptualization.

## Disclosures

The authors declare the following financial interests/personal relationships which may be considered as potential competing interests:

Mingan Choct reports financial support was provided by BASF SE. If there are other authors, they declare that they have no known competing financial interests or personal relationships that could have appeared to influence the work reported in this paper.
